# Meta-analysis of oral microbiome reveals sex-based diversity in biofilms during periodontitis

**DOI:** 10.1172/jci.insight.171311

**Published:** 2024-09-10

**Authors:** Rita Del Pinto, Claudio Ferri, Mario Giannoni, Fabio Cominelli, Theresa T. Pizarro, Davide Pietropaoli

**Affiliations:** 1 San Salvatore Hospital, Department of Clinical Medicine, Public Health, Life and Environmental Sciences, University of L’Aquila, L’Aquila, Italy.; 2 Department of Pathology, Case Western Reserve University School of Medicine, Cleveland, Ohio, USA.; 3 Oral DISeases and SYstemic interactions study group (ODISSY group), L’Aquila, Italy (detailed in Supplemental Acknowledgments).; 4 Center of Oral Diseases, Prevention and Translational Research, Dental Clinic, Department of Clinical Medicine, Public Health, Life and Environmental Sciences, University of L’Aquila, L’Aquila, Italy.; 5 Department of Medicine, Case Western Reserve University School of Medicine, Cleveland, Ohio, USA.

**Keywords:** Inflammation, Microbiology, Epidemiology

## Abstract

Sex is an often overlooked, yet compulsory, biological variable when performing biomedical research. Periodontitis is a common yet progressively debilitating chronic inflammatory disorder affecting the tissues supporting teeth that ultimately leads to tooth loss if left untreated. The incidence of periodontitis is sex biased, with increased prevalence in males compared with females but with unknown etiology. We performed a sex-specific meta-analysis using publicly available oral microbiome data from different sampling sites of patients with periodontitis and periodontally healthy controls; sex balance was established for each periodontal health condition. Our results show sex-based diversity in oral biofilms of individuals with periodontitis but not in their saliva, with increased abundance of several periodontal pathogens in subgingival plaques from females compared with males. We devised a quantitative measure, uniquely defined as the Microsexome Index (MSI), which indicates that sexual dimorphism in subgingival bacterial composition is a distinct feature of reduced microbial diversity during periodontitis but not under healthy conditions. In addition, we found that smoking exacerbates microsexome diversity in supragingival biofilms, particularly during periodontitis. Taken together, we provide insights regarding sex-based diversity in periodontitis, a disease with multiorgan associations, and provide the rationale for further mechanistic, diagnostic, and therapeutic studies.

## Abstract 2

The oral microbiome shows sex-based diversity in periodontitis patients, with increased abundance of several pathogens in females vs. males, that is exacerbated by smoking.

## Graphical abstract

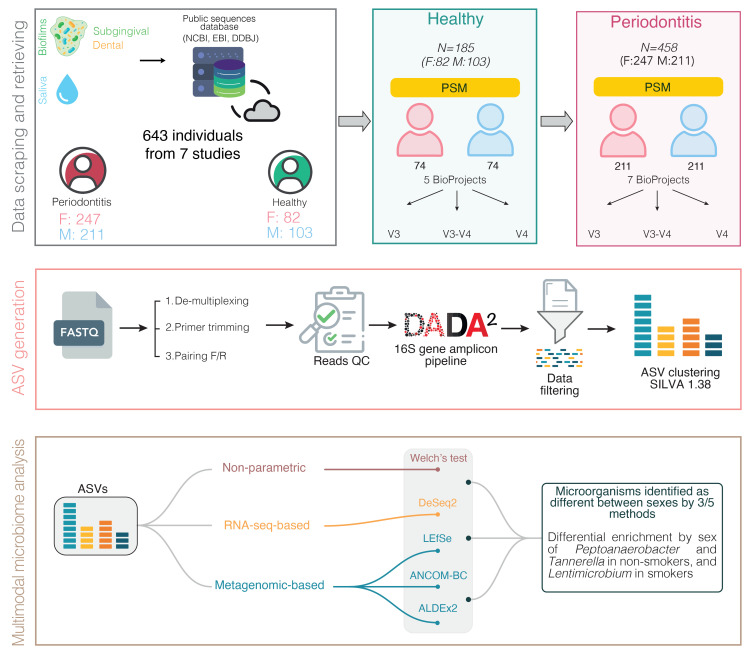

## Introduction

Evidence of sex-specific differences in gut microbiome composition, initially referred to as the microgenderome (1–3) and more recently proposed as the microsexome (4) or micro-sex/-genderome (5), together with findings of epidemiological and phenotypic differences between sexes in several chronic diseases (6–8), strongly supports the concept of sexual divergence in microbial ecosystems as a biologically plausible driver, or modifier, of disease states. Indeed, sex-specific studies of the microbiome during metabolic and autoimmune disorders, as well as in cancer, have focused on gut bacterial composition (1, 3, 9), the majority of which, thus far, have been conducted from sampling of the lower gastrointestinal tract (3, 9–12). These include meta-analyses of fecal metagenomes (9, 11), wherein unique microbial signatures are found to be associated with a variety of chronic diseases. For example, enrichment of pathogenic bacteria, such as *Fusobacterium* and *Porphyromonas*, are reported to characterize colorectal cancer (9), while depletion of bacteria associated with health, specifically butyrate-producing *Clostridiales*, distinguishes patients with inflammatory bowel disease (IBD) from healthy controls (11). Notably, despite their anatomical and functional connections to the gut, oral ecosystems have not been extensively studied, although local microbial composition, distribution, and organization in functional microniches have been implicated in diseases of the mouth, such as oral cancers (13) and periodontitis (14, 15).

Periodontitis is a chronic, progressively debilitating inflammatory disease that affects the integrity of tissues supporting the teeth, and it ultimately leads to tooth loss if left untreated. Indeed, periodontitis represents a huge global healthcare burden. In 2019, over 1 billion people were affected by severe periodontitis, representing the sixth most prevalent disease worldwide (16, 17). Importantly, clear associations with mortality and other diseases that also impose major healthcare and economic burden, including cancer and cardiovascular disease, have been established with periodontitis (18, 19). The direct dental treatment expenditure is estimated at US $298 billion per year (approximately 4.6% of global health expenditure), with an indirect cost amounting to US $144 billion yearly, making periodontitis one of the 10 most costly diseases globally (20). While a precise causal role for microbes has not yet been established for periodontitis, local microbial composition and its interactions with the immune system are thought to participate in disease pathogenesis and progression (21). Paramount to our study, males display a higher prevalence of periodontitis compared with females, with hormonal fluctuations during women’s life stages linked to increased disease susceptibility and severity (22, 23). The precise etiology of these sex differences in periodontitis, however, is currently unknown.

The central hypothesis of our study is that sexual dimorphism observed in periodontitis is driven, in large part, by differences in the oral microbiome and its interactions with local host mucosal immune responses. However, no studies to date have determined whether the microbiome from different oral sampling sites in health and during periodontal disease is differently shaped between sexes. Such sexual dimorphism in dysbiotic microbial communities could modify host immunity in a sex-specific manner (24) that can, in turn, differentially affect disease phenotype during the progression of life stages in women compared with men.

Herein, using a combination of multimodal, cutting-edge approaches for microbiome analysis, we report the observation of microsexome dysbiosis in oral biofilms during periodontitis that is not present in healthy individuals. Specifically, *Synergistota* and *Spirochaetota* are found to be enriched in dental plaque, and *Firmicutes* enriched in subgingival plaque, of females and males with periodontitis, respectively. Smoking exacerbates the sex-based divergence in dental plaque composition, even more so in individuals with, compared with those without, periodontitis; however, uniquely during periodontitis, sex-based differences in subgingival plaque composition occur regardless of smoking habits. Furthermore, we describe the noted sexual dimorphism in bacterial composition by devising a quantitative measure of sex-specific diversity that we define as the Microsexome Index (MSI), which in the present study is higher in females compared with males, revealing that, in the case of periodontitis, the disease-typical sex separation in microbial features occurs in parallel with reduced bacterial diversity. As such, the evidence presented in this study provides a conceptual advance to the literature by reporting a previously undocumented sex difference in the oral microbiome — specifically in supragingival and subgingival biofilms, but not in saliva — that is affected by smoking status in patients with periodontitis. At the same time, our findings raise provocative questions concerning the potential role of the microsexome not only in periodontitis but across several disease settings, including whether other environmental factors, aside from smoking, that can differentially modulate microbiome composition in a sex-dependent manner to affect disease. This information will prompt future studies to examine specific mechanisms attributed to the microbiome in the pathogenesis of sex-biased disorders, such as periodontitis, which will lead to improved diagnostic and precision-based therapeutic approaches to treat male versus female patients.

## Results

### Data features included in the meta-analysis.

In this meta-analysis, a total of 7 studies with publicly available data and metadata are included that use 16S amplicon sequencing (specifically, FASTQ or FASTA) of oral samples (e.g., saliva, dental plaque, or subgingival plaque) to characterize microbial features in periodontally healthy individuals and during periodontitis (Supplemental Figure 1; supplemental material available online with this article; https://doi.org/10.1172/jci.insight.171311DS1). Of the 7 studies, 4 assessed V3-V4 regions, 2 reported on the V4 region, and 1 examined the V3 region (Supplemental Table 1). Five studies were conducted on both periodontally healthy individuals and patients with periodontitis, while 2 included only patients with periodontitis (PRJNA324274, PRJNA477241), for a total of 643 participants (*N* = 458 with periodontitis; *N* = 185 healthy controls). Only 3 bioprojects reported race/ethnicity as metadata (PRJNA773202, PRJNA774299, PRJNA774981), and all participants in these 3 studies were defined as Caucasian. In terms of sampling sites, 2 studies (PRJEB6047 and PRJNA321534) assessed the microbiome in 2 oral mucosal sites — dental and subgingival plaques (PRJEB6047) and dental plaque and saliva (PRJNA321534) — while the remaining 5 studies examined microbial composition of a single sampling site (Supplemental Table 1). Information on disease severity was only reported by 3 bioprojects (PRJNA773202, subgingival plaque; PRJNA774299 and PRJNA774981, saliva), with most participants affected by severe disease and the remaining from moderate disease. Only 2 saliva-based bioprojects (PRJNA774299, PRJNA774981) reported on extent of disease (i.e., generalized and localized periodontitis). Within each periodontal-health condition, we applied 1:1 propensity score matching (PSM) for the variable sex to derive 2 separate cohorts of individuals with periodontitis (*N* = 422; Supplemental Table 2) and with healthy periodontium (*N* = 148; Supplemental Table 3), in which males and females are equally distributed. In addition, males and females within each cohort did not differ for age and sampling sites.

### Detection and management of potential confounders.

**Figure 1:**
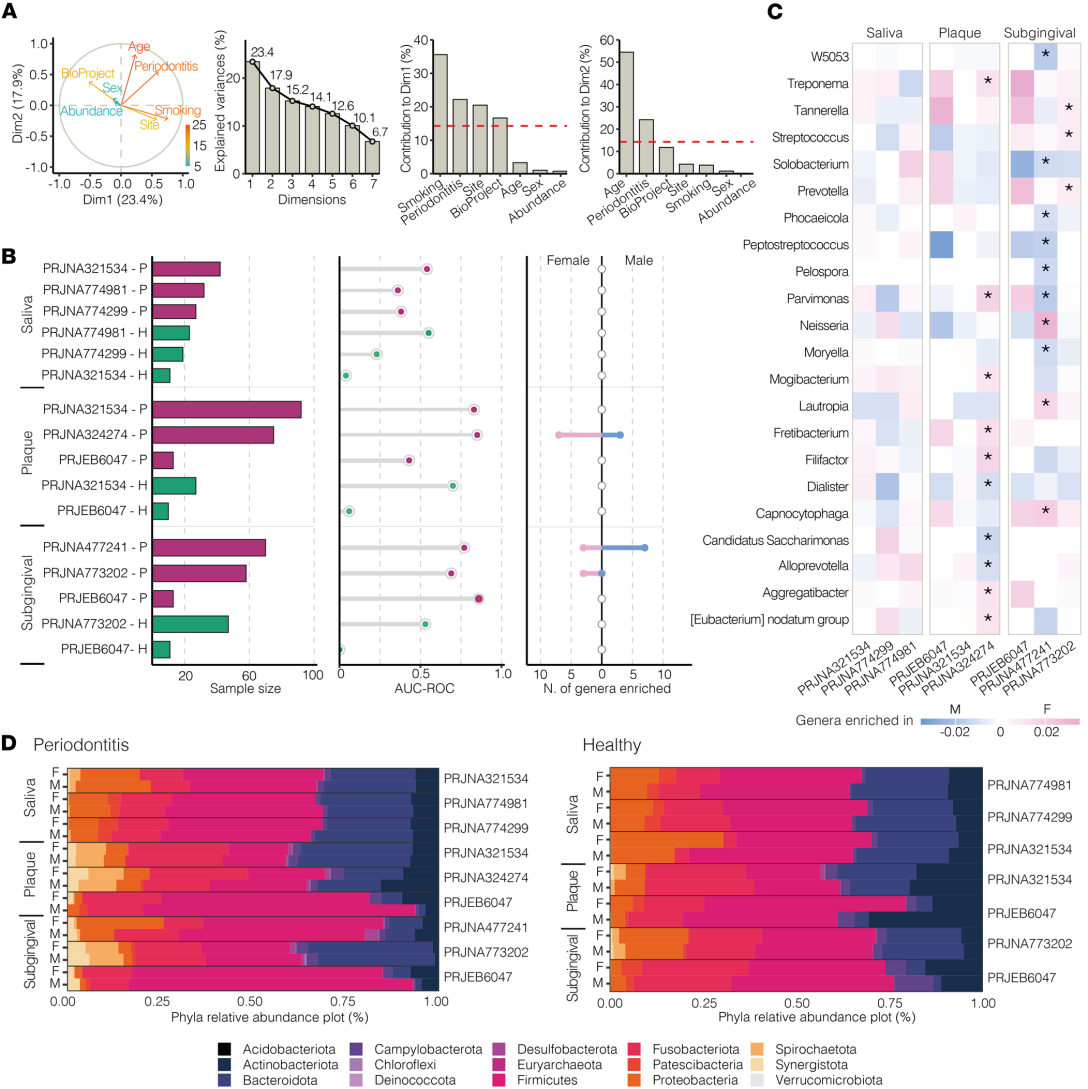
Overview of main determinants driving differential enrichment of microbial composition between sexes. (**A**) Variable correlation plot, eigenvalues bar plot, and contribution of variables to PCA dimensions 1 and 2 (Dim1 and Dim2, respectively) (left to right). Arrows (left panel) show color gradient from blue to red, representing low to high importance. Red dashed lines (far right panels) indicate expected average contribution of variables to PCA Dim1 and Dim2. Smoking, periodontitis, (sampling) site, and BioProject are identified variables contributing to the most substantial variance in data set. (**B**) Sample size for each site and periodontal condition (left). Studies on *y* axis grouped by disease status (periodontitis [P], violet; healthy controls [H], green) and ordered by decreasing sample size (top to bottom) within each site. AUC-ROC for genus-level random forest (RF) classifiers (middle). Classification of saliva samples was poor for both periodontal conditions (periodontitis: AUC = 0.60; healthy periodontium: AUC = 0.63). Plaque microbiome distinguishes females (Fs) from males (Ms) with healthy periodontium for 1 data set (AUC = 0.70), while biofilm microbiome (dental, subgingival plaques) distinguishes Fs from Ms during periodontitis with high to very high ability to classify in 4 data sets. Number of genera enriched in Fs (pink) and Ms (blue) for each study; *q < 0.05 by Welch’s test, Benjamini-Hochberg FDR correction (right). Only biofilm-associated microorganisms during periodontitis show differences between sexes. (**C**) Qualitative details of sex-based differential enrichment in genera during periodontitis; *q < 0.05 by Welch’s test, Benjamini-Hochberg FDR correction. (**D**) Relative abundance plot of phyla by sampling site in Fs and Ms during periodontitis (left) and in healthy individuals (right).

The aim of this meta-analysis was to determine whether any sexual dimorphism exists in the composition of the oral microbiome from periodontally healthy individuals and during periodontitis across the 7 included studies. Thus, before performing the sex-specific meta-analysis, we quantified the effects of study-associated heterogeneity on oral microbiome composition (Figure 1A and Supplemental Figure 2). Using principal component analysis (PCA) (Figure 1A), we found that smoking, disease status, sampling site, BioProject, and age were the variables that contributed the most to the variance in the data analyzed.

Given the predominant effect of the BioProject and sampling site factors on microbial composition regarding variance explained by sex (Figure 1A and Supplemental Figure 2), we used a combination of approaches to optimize the agreement level and consistency of results across studies according to the literature (25) (i.e., Welch’s test, DeSeq2, linear discriminant analysis effect size [LEfSe], ANCOM-BC, and ALDEx2; refs. 26–30). In parallel, we performed stratified analyses based on disease status, sampling sites, and exposure or nonexposure to smoking, as reported in the following sections. Interestingly, age was found to be relevant in shaping microbial composition in periodontally healthy individuals but less so during periodontitis (Supplemental Figure 2). Thus, metaregression based on age was performed.

### Sex-based differences exist in biofilm composition during periodontitis.

To test whether any sexual dimorphism in the oral microbiome exists during periodontitis and in periodontally healthy individuals, we built genus-level random forest (RF) classifiers to define female and male individuals within each site- and condition-specific data set and compared the resulting area under the receiver operating characteristic curves (AUC-ROC) across data sets (Figure 1B). While plaque microbiome classified periodontally healthy females from males in only 1 data set (AUC = 0.70), we were able to distinguish female from male patients with periodontitis in 4 data sets (AUC > 0.75, high to very high classifiability), all of which were biofilm specific (dental and subgingival plaques) (Figure 1B). Notably, 2 of the 4 periodontitis-specific data sets show very high classifiability (AUC ≥ 0.85). Sex-specific bacterial enrichment limited to subgingival and dental plaques during periodontitis is observed that involves up to 22 genera, depending on the data set (Figure 1C). Consistent with this finding, sex-based differences at the phylum level within data sets are evident in biofilms, but not in saliva, especially during periodontitis (Figure 1D). Thus, while salivary microbiome does not show any sex-based microbial differences in either healthy or disease states, biofilm composition exhibits sexual dimorphism during periodontitis.

### Meta-analysis of periodontitis studies reveals disease-associated, sex-specific enrichment in biofilm composition that efficiently predicts sex.

**Figure 2:**
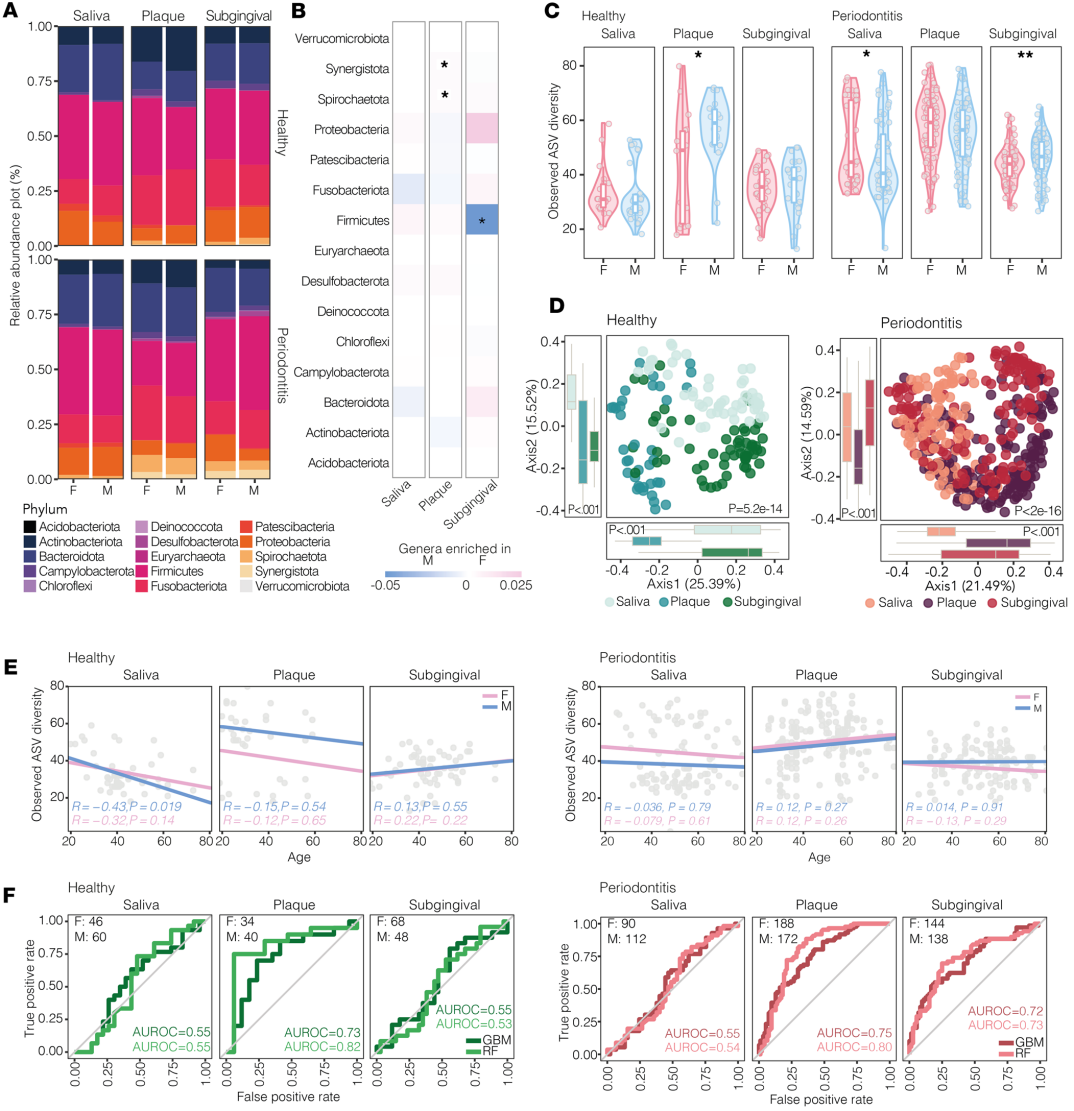
Meta-analysis reveals disease-associated sex-specific enrichment of biofilm composition from dental/subgingival plaques and reliably predicts sex during periodontitis. (**A**) Relative abundance plot of global phyla by (sampling) site in females (Fs) and males (Ms) without (top) and with (bottom) periodontitis from 7 included studies. (**B**) Heatmap showing log_10_ mean difference between sexes at phylum level across sites during periodontitis, with significant sex-specific enrichment indicated by asterisks (*q < 0.05 by Welch’s test, Benjamini-Hochberg FDR correction). Mean difference of phyla shown by sex, with pink/blue indicating enrichment in Fs and Ms, respectively. Opacity ranges from mean difference of 0.05 (M) and 0.025 (F) to 0 (white), wherein white indicates no difference between sexes. (**C**) α-Diversity (observed ASVs) by sex in healthy controls (left) and during periodontitis (right) across sites show reduced diversity in Fs at level of dental (healthy) and subgingival (periodontitis) biofilms; *q < 0.05, **q < 0.01 between sexes by Wilcoxon rank-sum test. (**D**) β-Diversity of samples from all 7 studies based on Bray–Curtis distance indicate strong influence of sampling. Box plots show samples projected onto first 2 principal components broken down by site; *P* values by Kruskal-Wallis test. (**E**) α-Diversity of biofilms does not correlate with age in both sexes. Salivary α-diversity shows weak, negative correlation with age in periodontally healthy Ms after calculation of Pearson’s correlation coefficient. (**F**) AUC-ROC curves built with RF and gradient boosting machine (GBM) show predictive power of microbiome to predict sex for each sampling site and periodontal condition. Different from salivary microbiome, biofilm-associated microbiome correctly classifies Ms versus Fs during periodontitis, while in periodontally healthy individuals, classification of sex was poor for both salivary and subgingival microbiomes.

After pooling data sets, sex-based differences in phyla relative abundance are more visible in biofilm composition (dental plaque in healthy individuals; dental and subgingival plaques during periodontitis) than in saliva, particularly during disease (Figure 2A). In agreement with these data, significant sex-specific enrichment of phyla in biofilms is found during periodontitis but not in healthy periodontium, with *Synergistota* and *Spirochaetota* enriched in the dental plaque of females and *Firmicutes* enriched in the subgingival plaque of males affected by disease (Figure 2B and Supplemental Figure 3).

Microbial richness in saliva and in subgingival plaque from periodontally healthy individuals and during periodontitis shows similar sex-based trends, which only became significant during disease (Figure 2C). Interestingly, reduced bacterial richness is observed in females at the level of dental (healthy) and subgingival (periodontitis) biofilms that is consistent with results showing an increased richness in saliva composition of females during periodontitis (Figure 2C); this is in agreement with the strong influence of sampling site on the microbiome (Figure 2D) and is independent of age (Figure 2E).

In general, the composition of biofilms, but not of saliva, is able to classify sex in periodontally healthy individuals (dental plaque) and during periodontitis (dental and subgingival plaques) (Figure 2F). In periodontally healthy individuals, classification of sex is poor for both salivary and subgingival microbiomes. Thus, subgingival microbiome does exhibit a sexual dimorphism during periodontitis, and this is not observed in individuals with a healthy periodontium.

### Smoking exacerbates sexual dimorphism in dental plaque, but sexual dimorphism in subgingival plaque is unique to periodontitis regardless of smoking habits.

**Figure 3:**
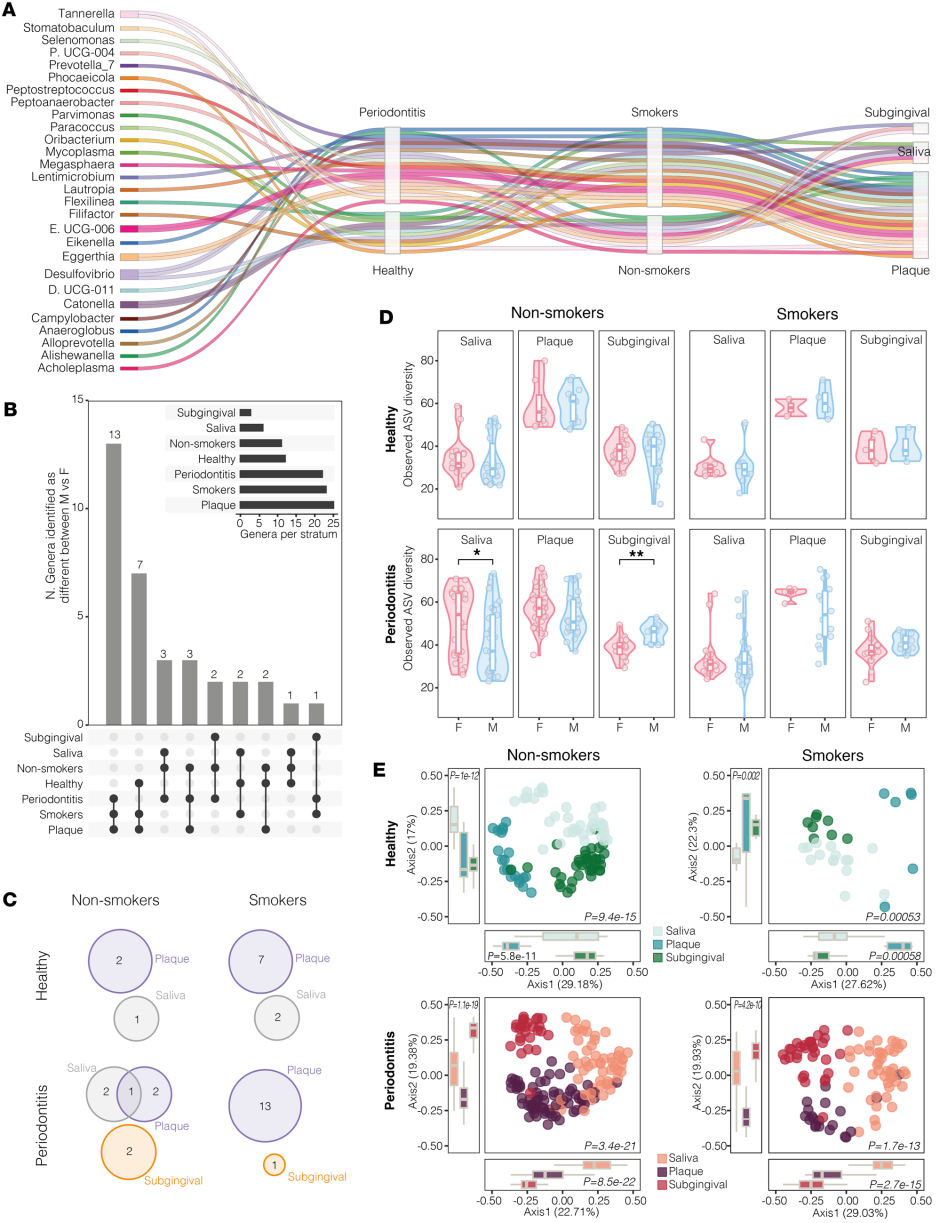
Smoking exacerbates sexual dimorphism in dental plaque composition but, in subgingival plaque, is independent of smoking and unique to periodontitis. Individuals with unknown smoking status were excluded. (**A**) Alluvial plot showing sexually dimorphic genera and their position across reported axes strata. Splines correspond to genera identified as consistently different between males (Ms) and females (Fs) using multiple differential abundance (DA) analysis methods (see Supplemental Figure 4 for DA outputs). Axes correspond to periodontal condition, smoking habits, and (sampling) site; each genus is indicated by a specific color. (**B**) Multiple response analysis, reporting number of diverse genera by sex across combinations of periodontal condition, smoking habits, and site (black dots, connecting bars). More diverse genera based on sex are found in supragingival plaque of smokers during periodontitis. Summary of number of different genera by sex in corresponding conditions. (**C**) Venn diagrams show only 1 genus (i.e., *Desulphovibrio*) as consistently different between sexes in 2 different sites, saliva and supragingival plaque, of nonsmokers with periodontitis. Differential enrichment of subgingival plaque by sex is only evident during periodontitis, independent of smoking. (**D**) α-Diversity stratified by sex across periodontal condition and smoking habits show sex-based differences in α-diversity in saliva and subgingival biofilms from nonsmokers with periodontitis. *P<0.05, **P<0.01 by Wilcoxon rank-sum test. (**E**) β-Diversity by smoking status in each disease condition. Segregation patterns are affected by site and smoking habits, determined by Kruskal-Wallis test.

We next examined sex-based microbial differential abundance (DA) across sampling sites during health and disease as well as in the presence and absence of exposure to tobacco smoke. To this end, we integrated results from multiple methodologies exploiting diverse approaches to microbiome data for DA analysis; it allowed us to assess the reliability and consistency of findings, based on the consensus level between methodologies, and to provide more context to improve overall interpretation, according to literature (25) (Supplemental Figure 4). Our results show that a total of 28 genera are found to be differentially abundant between females and males across combinations of periodontal conditions (disease/health) with smoking habits (smokers/nonsmokers) and sampling sites (saliva, dental plaque and subgingival plaque) (Figure 3A). Two genera (*Tannerella* and *Desulphovibrio*) are differentially abundant between sexes in both periodontal conditions but in different sites and/or smoking habits. Globally, we found more sexually diverse genera in the dental plaque of smokers during periodontitis (*N* = 13), followed by periodontally healthy individuals (*N* = 7) (Figure 3, A–C). At the level of subgingival plaque, differential enrichment by sex is only evident during periodontitis, regardless of smoking status (*Peptoanaerobacter* and *Tannerella* in nonsmokers, *Lentimicrobium* in smokers) (Figure 3, A–C).

Preliminary to this, the analysis of α-diversity indicates that no significant sex-based differences exist in the microbial richness across all sampling sites in healthy individuals, independent of smoking habits (Figure 3D). Conversely, in nonsmokers with periodontitis, the subgingival microbiome shows increased richness in males, while saliva composition indicates increased richness in females (Figure 3D and Supplemental Figure 5), consistent with the influence of sampling sites and smoking habits on segregation patterns (Figure 3E).

Taken together, smoking exacerbates sexual dimorphism in dental plaque composition, especially during periodontitis. In parallel, independent of exposure to tobacco smoke, the presence of periodontitis has an effect on sexual dimorphism of the subgingival plaque ecosystem, which is not observed under normal healthy conditions.

### The MSI as a measure of sex-specific diversity.

We then asked whether sexual dimorphism in microbiome composition could be summarized in a quantitative measure of sex-specific diversity that may be useful to explore correlations with overall bacterial richness. This question was answered with an approach modified from Gevers et al. (31), by which we applied the analysis to the subgingival microbial composition of nonsmokers, given the relevance of smoking to oral biofilms.

**Figure 4:**
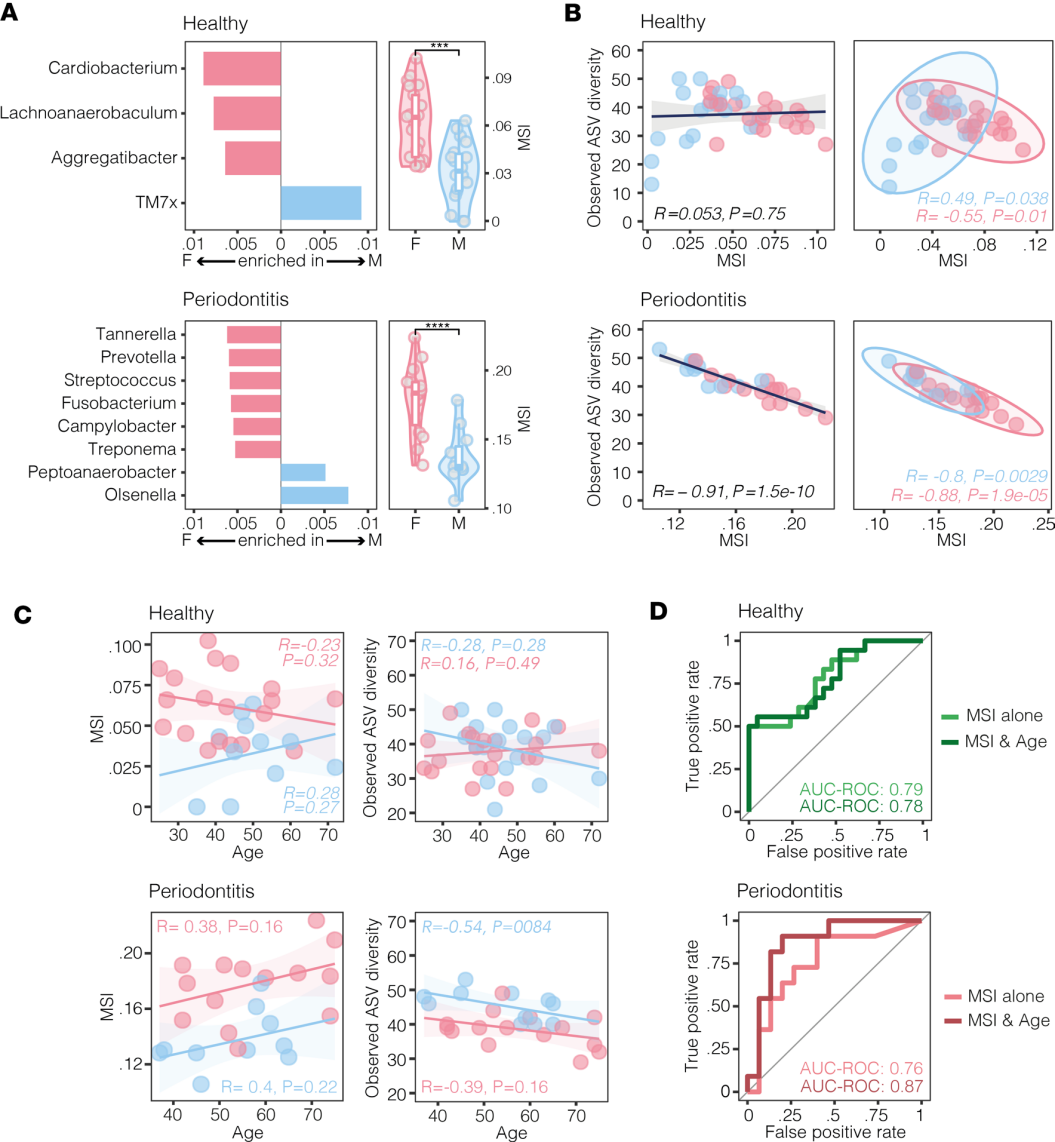
The Microsexome Index (MSI) represents a quantitative measure of sex-specific diversity. In order to reduce bias from smoking, MSI was calculated based on subgingival microbiome composition of nonsmokers, excluding individuals with either unknown smoking history or who reported being smokers. (**A**) Genera differentially abundant between sexes in periodontally healthy individuals and during periodontitis, after applying FDR to 2-tailed *t* test (31), were used to compute MSI, a summary statistic of observed sex-specific microbial diversity wherein values different from zero indicate increased sex-specific enrichment (left). Violin plots show statistical difference in MSI between sexes by periodontal condition, indicating MSI as a summary measure of sex-predictive power of subgingival microbiome (right*)*. ***P < 0.001; ****P < 0.0001 by Wilcoxon rank-sum test. TM7x, *Saccharibacteria*. (**B**) Relationship of MSI with subgingival microbial richness overall (left) and by sex (right), calculated by applying Pearson’s correlation coefficient. Different from periodontally healthy controls (top left), MSI shows significant, inverse relationship with subgingival microbial richness during periodontitis (bottom left). When dissecting information by sex (right), periodontally healthy males uniquely show a significant, direct correlation of MSI with richness. (**C**) Relationship of MSI and subgingival microbial richness with age by periodontal condition using Pearson’s correlation coefficient, with evidence of a trend toward increased MSI and parallel decrease in richness with age, not observed in healthy controls. (**D**) Consistent with this, MSI reliably predicts sex in both periodontal conditions, but addition of age increases predictive accuracy only during periodontitis. Data visualization refers to healthy Fs (*N* = 21), healthy Ms (*N* = 18), periodontitis Fs (*N* = 15), and periodontitis Ms (*N* = 11). AUC-ROC with RF is applied.

First, analysis of the sex-specific compositional nature of the subgingival environment shows a differential enrichment in taxa between females and males, with double the number of differentially enriched genera during periodontitis (*N* = 8) compared with periodontally healthy individuals (*N* = 4) (Figure 4A). In fact, a female-specific richness is observed for 6 taxa during periodontitis versus 3 taxa in healthy controls, while 2 taxa are male specific that are enriched during periodontitis versus only 1 taxon in healthy controls (Figure 4A). We then calculated, for both periodontal conditions, the Log of the ratio of the relative abundance +1 of organisms enriched in females over those enriched in males, hereafter referred to as the MSI. In theory, the MSI represents a summary statistic of the observed sex-specific microbial diversity, with increased sex-specific enrichment for values different from zero. The MSI shows a strong negative correlation with species richness in periodontitis but not in periodontally healthy individuals (Figure 4B), demonstrating that sexual dimorphism in bacterial composition is a distinct feature of reduced microbial diversity in the disease state but not under healthy conditions. Indeed, periodontally healthy males uniquely show a significant and direct correlation of the MSI with microbial richness. In line with this concept, during periodontitis — but not in healthy controls — the MSI tends to increase with age in parallel with a decrease in overall microbial richness (Figure 4C). Also consistent with this finding, the MSI is able to accurately predict sex in both periodontal conditions, but with the additional consideration of age, the MSI increases the predictive accuracy only during periodontitis (Figure 4D). Taken together, the MSI represents a unique identifier of sex-based microbial diversity.

## Discussion

The results of the present meta-analysis of oral metagenomes from individuals with and without periodontitis reveal microsexome diversity in biofilms during disease that is not paralleled by sex-specific salivary patterns, wherein females with periodontitis have an increased abundance of several periodontal pathogens in subgingival plaque compared with men. Disease-typical sex separation in microbial features occurs concomitantly with reduced bacterial diversity, as evidenced by the inverse relationship between the MSI and α-diversity. Smoking exacerbates disease-specific sexual dimorphism in the composition of dental biofilms, but separation by sex in subgingival plaque is found to be independent of smoking and unique to periodontitis.

Notably, no previous studies have addressed the microsexome question during periodontitis or, for that matter, in healthy periodontal conditions; furthermore, no attempts been made, to our knowledge, to investigate whether sex-specific oral microbiome features exist across differentially accessible oral sampling sites, specifically in biofilms and saliva. In addressing these knowledge gaps, we provide evidence that resorting to multiple analytical methods increases consistency and precision in these types of microbiome studies (25). In this setting, we introduce a unique identifier, the MSI, as a quantitative measure of sex-based diversity that occurs in a structured consortia of bacteria within the subgingival niche, at the interface with the host immune system. While the immunological implications of our findings are unknown, there is evidence that highly organized bacterial niches are major modulators of immune cells through several mechanisms, including genetic modulation (13), and that local inflammation during periodontitis can be passed on epigenetically to myeloid cells and determine maladaptive-trained myelopoiesis, linking periodontitis with systemic inflammatory diseases (19). In fact, we have recently shown a female-specific association between gingival bleeding, a hallmark feature of active inflammation during periodontitis, and metabolic syndrome, which is a cluster of metabolic risk factors underpinned by low-grade systemic inflammation (32). Understanding whether subgingival biofilm composition differs by sex in relation to periodontal health status might lead to more effective strategies against autoimmune diseases (33) and chronic inflammatory disorders, for which sex and host immunity play a role, including systemic lupus erythematosus (8), rheumatoid arthritis (7), and type 1 diabetes mellitus (6). This is even more intriguing when considering that, besides having a sexually divergent epidemiology (22), periodontitis is associated with a variety of systemic chronic inflammatory conditions (34), including IBD (35–37), which have also been mechanistically linked to the local microbial burden, gut dysbiosis (38), and immune activation (19).

The oral microsexome might also be of interest as a potential screening and/or monitoring tool for diseases of the lower gastrointestinal tract, such as IBD, celiac disease, and colorectal cancer. There is, in fact, the possibility for salivary microbiome composition to predict gastrointestinal and liver diseases (39, 40), and perturbations of oral biofilms during periodontitis have also been proven to affect microbial gut composition, leading to local dysbiosis (38). Recently, increased levels of *H*. *parainfluenzae* strains were reported to be detected in patients with Crohn’s disease and concomitant periodontal disease, with colonization of *H*. *parainfluenzae* eliciting strain-dependent intestinal inflammation in murine models of IBD (41). Whether a sex-specific approach could refine the diagnostic accuracy and provide a more mechanistic understanding underlying disease trajectories in settings wherein host-microbial interactions influence disease pathogenesis and progression is a topic that warrants further investigation.

As a hallmark feature of host immunosurveillance of, in this case, the oral cavity, serum antibodies against periodontal bacteria represent host-specific humoral responses by phenotypic expression of the microbial-immunological interface (42, 43). Interestingly, we found evidence of sexually dimorphic activation of the immune system, in terms of the presence of specific antibodies to periodontal bacteria, in a population with periodontitis, which is not observed in periodontally healthy individuals (Supplemental Figure 6 and Supplemental Table 4). Specifically, we document a positive, female-specific correlation between genus-ranked relative antibody abundance and subgingival microbial abundance at the same taxonomic level in individuals with periodontitis. Conversely, microbial and immunological features were correlated in both sexes among periodontally healthy controls, with no evidence of sexual dimorphism in regard to immune activation toward subgingival bacteria under healthy conditions (Supplemental Figure 6). This additional finding suggests that the sexually diverse subgingival enrichment during periodontitis might uniquely characterize immune activation in a sex-specific manner, a finding that is not observed in periodontally healthy individuals. Further research is required to confirm these findings.

Among the bacteria identified that contribute to subgingival microsexome diversity during periodontitis are *Peptoanaerobacter* and *Tannerella*, for which local and systemic virulence have been reported (44, 45). Previously described as more abundant in the subgingival biofilm composition of smokers with periodontitis (46), their sexually diverse abundance in nonsmoking, periodontopathic individuals suggests that sex differences in these pathogenic bacteria emerge in the absence of major external factors, such as smoking, known to favor their enrichment. Expanding on the observation that exposure to tobacco smoke promotes the creation of pathogen-rich biofilms, we found that smoking exacerbates sex-based separation in dental biofilm composition, especially during periodontitis. In addition, microsexome diversity in dental plaque of healthy smokers more often involves genera associated with periodontitis in nonsmokers (e.g., *Defluviitaleaceae*, *Desulfobulbaceae*) (38), thereby supporting previous evidence of a microbiome shift toward a typical disease-associated community enriched in anaerobic pathogens in periodontally healthy smokers (47). In the same line of thought, the strict anaerobic, periodontitis-associated bacterium, *Desulfovibrio* (48), here documented among the sexually diverse bacteria in healthy smokers and during periodontitis in nonsmokers, is reported to increase after exposure to lead, a heavy metal found in cigarette smoke (49).

This analysis does not include all published studies evaluating oral microbiome from patients with periodontitis, largely due to unavailable microbiome data and metadata from public repositories. The lack of metadata of interest — e.g., measures of periodontitis severity or therapeutic and/or contraceptive use of sex hormones — prevented any additional focused analysis on the included studies, as well as the possibility to run meta-analyses on different study types (e.g., metagenomic whole genome shotgun [WGS] studies). However, implementing microbiome data sharing and metadata availability will allow more detailed analyses in the future. It must also be noted that 16S rRNA gene data should not be considered less reliable than other microbiome data types, such as shotgun metagenomics, whose levels of sparsity and intersample variation may still be relevant, or even superior, as compared with 16S rRNA (25). Additionally, taxonomic resolution was limited to the genus level for concerns of technical interstudy batch effects, which could be implemented in future studies looking at microsexome diversity at the species level by means of last generation metagenomic classifiers. Despite the application of PSM to achieve balance in terms of sex, age, and sampling site, other variables that could not be accounted for prevent generalizability of our findings.

Importantly, it should also be pointed out that sex of study participants in the present meta-analysis was self-reported and not necessarily sex assigned at birth. As such, we acknowledge that there may be limitations to the study since the conclusions drawn, as they may relate to transgender individuals, are unknown. Furthermore, we use the term microsexome versus microgenderome throughout our study since we defined our patient cohorts by self-reported sex that is more restricted to biological characteristics versus gender, which, while encompassing a broader spectrum, generally reflects a social construct that includes characteristics beyond biological sex. Nonetheless, it can also not be ruled out that gender (and not only sex) can influence the microbiome, and factors such as diet, physical activity, antimicrobial exposure, and psychiatric comorbidity (4) have been proposed to impact the microbiome. In the last several years, research focused not only on the influence of sex but also of gender in disease processes is rapidly increasing and should glean important information in this intriguing area of investigation.

In conclusion, in the present study, by demonstrating microsexome diversity in the periodontal niche during periodontitis, we provide a better understanding of sexual dimorphism in a disease with multisystemic associations, possibly serving as the foundation for the implementation of minimally invasive diagnostic and monitoring tools within, and outside, the boundaries of periodontal disease; this may lead to successful advancements toward personalized and precision medicine. Future research is greatly needed regarding the study of microsexome diversity in the oral-gut axis, its mechanistic role in disease burden, progression and eventual phenotypic trajectories of disease, effect on known comorbidities, and any potential therapeutic implications.

## Methods

### Sex as a biological variable.

Female and male individuals were included in this study; see below for the application of PSM for the variable “sex” to obtain an equal distribution of females and males within each condition of interest.

### Data set collection.

The NCBI BioProject database (https://www.ncbi.nlm.nih.gov/bioproject/) was interrogated for studies that published oral metagenomic data of patients with periodontitis and healthy controls with the R library “rentrez” using the following terms: “periodontitis”, “periodontal”, or “periodontal disease*”. Project titles, descriptions and other information were exported into a spreadsheet for subsequent manual eligibility checking. Human metagenomic projects with participants reporting on, or assessed for, periodontal health status were admissible if raw 16S rRNA-gene amplicon sequencing data (FASTQ or FASTA) were publicly available, and if they were from saliva, dental plaque, or subgingival plaque samples with metadata indicating case (periodontitis) or control (healthy) status as well as biological sex for each sample. No restrictions were applied based on study design (e.g., cohort, interventional case–control or cross-sectional studies), geographical area, race/ethnicity, or periodontal disease activity. When available, demographic identity was collected as reported in the original studies. Non-English studies, review articles, nonhuman studies, case reports, studies on nonperiodontal specific conditions/diseases, intervention trials without preintervention data of interest, nonmetagenomic or archeological studies, and studies without publicly unavailable data sets/metadata were excluded, as were those reporting on recent (<6 months) surgical/nonsurgical periodontal treatment as well as explicit current or recent (<1 month) antibiotic use. Data and matched patient metadata were downloaded from the sequence read archive (SRA) repository of the European Nucleotide Archive (ENA) (https://www.ebi.ac.uk/ena/browser/home). We did not include studies requiring either additional ethics committee approval, authorizations for access, or personal communication with authors.

On December 27, 2021, 466 studies were admissible, of which 141 met the inclusion criteria and 108 had both SRA data and metadata (*N* = 10,618 biosamples). Since different sequencing technologies could have been used among studies, biosamples were screened for duplicate (*N* = 317), nonamplicon (*N* = 2,341), and non-Illumina (*N* = 1,387) sequences. The remaining 6,573 biosamples from 62 BioProjects underwent further 1-by-1 in-depth evaluation for admissibility, and relative metadata of interest were extracted. After applying specified inclusion and exclusion criteria to the 704 sequences and relative metadata, a total of 643 sequences from 7 BioProjects were used for analysis in the present study (see Supplemental Figure 1 for data reduction). Thus, 2 distinct cohorts of patients with periodontitis (*N* = 422) and healthy individuals (*N* = 148) were generated, in which 1:1 PSM for the variable “sex” was applied to obtain an equal distribution of males and females within each condition.

Bioinformatics were implemented in R (version 4.1.2) in a dedicated computer-optimized virtual machine on Google Compute Engine (C2-standard-30, 30 vCPU/128GB RAM, Ubuntu 18.04 LTS).

### 16S rRNA preprocessing.

Raw data were downloaded and processed through our in-house 16S rRNA processing pipeline. Since taxonomic classification might differ when targeting different hypervariable regions of the 16S rRNA (50), and given the consistently good performance of V3-V4 regions over other hypervariable regions in the identification of taxa (50, 51), only V3-V4 regions were selected for the analysis. Of the 7 included studies, 4 assessed V3-V4 regions, 2 reported on the V4 region, and 1 examined the V3 region.

Sequences were processed using the DADA2 pipeline (52). Specifically, primers were truncated according to the length of primers, as specified in the original paper when available; otherwise, trimming was performed at 20 bp for the forward and reverse ends (11). After visual inspection of sequence readings, sequences were quality filtered by truncating at the first base with a quality score Q < 25 (Supplemental Table 1) (11). Maximum number of expected errors (maxEE) and quality scores (truncQ) were set at integer 2. Amplicon sequence variants (ASVs) were assigned a taxonomy using the SILVA release 138 database (53). For each data set, we removed samples with fewer than 100 reads, ASVs with fewer than 10 reads, and ASVs that were present in less than 1% of samples within a study (11). We calculated the relative abundance of each ASV by dividing its value by the total reads per sample using a specific R library (54). Taxonomy table, ASVs, and metadata were condensed in a single phyloseq object and used for further analyses (55).

### Microbiome community analyses.

Based on the noncollapsed 100% ASV-level relative abundances, α-diversities expressed by the Shannon index were calculated. The average abundance of each phylum or genus was calculated as the mean of their respective average values. For the calculation of genus abundance, patients with zero abundance were excluded from the computation.

### Confounder analysis.

Although 1:1 PSM generated sex-balanced cohorts of periodontitis and healthy male and female individuals, who were comparable for age and sampling sites, other approaches were also adopted to control for major confounders. To quantify the effect of potential confounding factors relative to the effect of sex on single microbial features, 1-way ANOVA on rank-transformed data was performed (56). Rank transformation was performed to account for non-Gaussian distribution of microbiome abundance data. Using a generalized linear model (GLM), the association of log_10_ normalized phyla abundance with selected metadata (sex, age, smoking status, sampling site, study, and library size) were assessed. Age was used as a continuous variable. Then, total variance within single microbial phyla was compared with the variance explained by sex and the variance explained by possible confounding factors (i.e., study and sampling site) in relation to a linear model, where both sex and the putative confounding factor were included as explanatory variables for phyla abundance. Stratified analyses, based on sampling sites and smoking status, were also performed.

### The MSI.

The MSI was calculated as a summary statistic of sex-specific subgingival microbial diversity in nonsmokers with and without periodontitis, using methods modified from Gevers et al. (31). Adding to this methodology, we examined whether a quantitative measure of sex-specific diversity could be derived in periodontitis versus healthy gums that could dissect, in a sex-specific manner, the dysbiotic state and reduced bacterial richness observed during disease. In order to limit the influence of external factors on local flora, the MSI was calculated only on subgingival samples of nonsmokers, thereby excluding individuals with unknown smoking history or those who reported being smokers.

First, relative abundance of subgingival genera was derived using log_10_+1 normalization. Sex-specific genus enrichment in healthy and periodontitis nonsmokers was assessed applying FDR to 2-tailed *t* test, as specified elsewhere (31). Subsequently, the MSI relative to subgingival flora was calculated as the logarithm of the ratio between the relative abundance sum of female-enriched taxonomic groups + 1 and the relative abundance sum of the male-enriched taxonomic groups +1


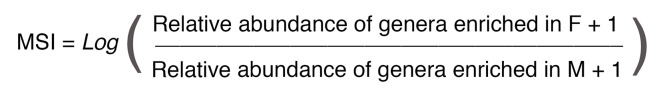
Equation 1

Relative abundancies were transformed to account for data sets containing zeroes. Thus, the MSI ranges from negative values (indicating male-sex richness) to positive values (indicating female-sex richness), with zero corresponding to the absence of sexual dimorphism in taxonomic richness. We then assessed the presence of any correlation of the MSI with Shannon α-diversity.

### Statistics.

DA analysis was used to identify sex-specific microbiome biomarkers in healthy controls and patients with periodontitis. To this end, ASVs were agglomerated either to phyla or genus, as appropriate, and multiple approaches — based on diverse DA methods, accounting for compositionality of microbiome data or relative abundances — were combined to increase the robustness of findings and to provide more context to improve their biological interpretation, according to the literature (25, 57). Specifically, Welch’s 2-tailed *t* test (26), DeSeq2 (27), LEfSe (28), ANCOM-BC (29), and ALDEx2 (30) were implemented using specific R libraries in the Microbiome package (54, 58). Visualizing data at multiple levels of taxonomic hierarchy offers insights into both broad patterns and finer details within the microbiome (57). Thus, analyses at the phylum level were performed to provide an overview of the major microbial groups that are differentially abundant between sexes, and those at the genus level were conducted to offer more detail of the biologically related microbial communities potentially driving the differences observed at higher taxonomic levels. For the DA testing, we created a custom R script, mainly implemented with the library MicrobiomeMarker (58), which allows building a unified toolbox for microbiome biomarker discovery by integrating existing widely used DA methods (59). Analyses were performed on the phyloseq object described earlier. Detailed description and script settings for each DA method are reported in Supplemental Table 5 and in the Supplemental Methods. Our significance cutoff was set to an α-value of 0.05, and FDR adjusted *P* values using Benjamini-Hochberg correction were used as appropriate (i.e., the correction does not apply to methods that do not generate *P* values, namely LEfSe) (60). Based on these specifications, microorganisms were considered as differentially abundant between sexes in case of agreement of at least 3 methods.

To perform supervised classification of males and females based on sex-specific microbial features within each data set, 2 approaches were used: (a) RF and (b) gradient boosting machine (GBM). Classifiers were implemented in the “caret” package using 3-times 10-fold cross-validation. In this way, repeated 10-fold cross-validation was performed 3 times, and the models that obtained the best results according to their AUC-ROCs were reported. Besides the overall cross-validation prediction, the discriminatory power of classifiers in terms of their accuracy, specificity, and sensitivity was calculated using the MLeval package.

### Study approval.

As an analysis of existing, deidentified data, IRB approval was deemed exempt.

### Data availability.

Raw sequencing data for each study can be accessed as described earlier. Raw processed ASV tables can be accessed in GitHub space, available at http://github.com/PietropaoliLab (commit ID: efbcf6d48d03dd8be22f6b68178d7d55c8066bf6). All other relevant data supporting findings of this study are available in the present manuscript and Supporting Data Values or upon request to the corresponding authors.

## Author contributions

RDP conceptualized the study, designed/performed experiments, analyzed/interpreted results, and wrote the manuscript. CF, MG, and FC provided resources and valuable input to the manuscript. TTP contributed to the study concept, interpreted results, provided resources, and edited and finalized the manuscript. DP conceptualized the study, designed/performed experiments, analyzed/interpreted results, and wrote the manuscript.

## Supplementary Material

Supplemental data

Supporting data values

## Version 1 09/10/2024

Electronic publication
