# Supplementary material for: Meta-analysis of oral microbiome reveals sex-based diversity in biofilms during periodontitis
Source: JCI Insight. 2024 Sep 10;9(17):e171311. doi: 10.1172/jci.insight.171311 (PMC11385077; doi:10.1172/jci.insight.171311)
Supplement: Supplemental data [file jciinsight-9-171311-s261.pdf]

Meta-analysis of oral microbiome reveals sex-based diversity in biofilms during periodontitis

Rita Del Pinto[1,2,3], Claudio Ferri[1,3], Mario Giannoni[3,4], Fabio Cominelli[5], Theresa T. Pizarro*[2], and Davide Pietropaoli*[3,4,5]

*Shared senior authorship

[1]San Salvatore Hospital, Department of Clinical Medicine, Public Health, Life and Environmental Sciences, University of L'Aquila, L'Aquila, Italy

[2]Department of Pathology, Case Western Reserve University School of Medicine, Cleveland, OH, USA

[3]Oral DISeases and SYstemic interactions study group (ODISSY group), L'Aquila, Italy

[4]Center of Oral Diseases, Prevention and Translational Research, Dental Clinic, Department of Clinical Medicine, Public Health, Life and Environmental Sciences, University of L'Aquila, L'Aquila, Italy

[5]Department of Medicine, Case Western Reserve University School of Medicine, Cleveland, OH, USA

SUPPLEMENTAL MATERIAL

Extended Methods

Differential abundance methods. According to the literature(1, 2), diverse differential analysis (DA) methods, accounting for compositionality of microbiome data or relative abundances, were combined to increase robustness of findings and provide more context to improve overall interpretation. These methods are reported below. A

custom R script, mainly implemented with the library MicrobiomeMarker (3), was created to build a unified toolbox for microbiome biomarker discovery by integrating these methods(4). Detailed script settings for each DA method are reported in **Supplementary Table 5**.

Welch's t-test. We applied total sum scaling normalization to the phyloseq object and then performed an unpaired two-tailed Welch's t-test for each taxa to compare sexes within each periodontal health condition. Benjamini-Hochberg correction was applied to correct resulting *P*-values for multiple testing. A *P*-value less than 0.05 was considered significant.

LEfSe. With LEfSe (Linear discriminant analysis Effect Size), we standardized data using total sum scaling, dividing the count of each feature by total library size. Subsequently, we conducted a Kruskal-Wallis test (which, in our case of two groups, simplifies to the Wilcoxon rank-sum test) to detect potentially differing abundances of taxa (cutoff = 0.05). This was followed by Linear Discriminant Analysis (LDA) of class labels based on abundances to estimate effect sizes for significant features. Only features with LDA analysis scores scaled above the threshold of 2.0 (default) were identified as differentially abundant between sexes. No multiple-test correction was applied to the raw LEfSe output since only *P*-values of significant features with above-threshold LDA scores are provided by this tool.

DESeq2. We inputted the phyloseq object into the DESeq2 function with default settings, establishing estimation of size factors to ‘poscounts’, which accounts for features missing in at least one sample. The function underwent three main steps: it first estimated size factors to normalize library sizes in a model-based manner; then, it estimated dispersions using the negative binomial likelihood for each feature,

followed by shrinkage towards the default trendline via empirical Bayes; finally, it fit each feature to the specified class groupings using negative binomial generalized linear models and conducted hypothesis testing with the default Wald test. We obtained resulting Benjamini-Hochberg FDR-corrected *P*-values using the results function.

ANCOM-BC. Analysis of Compositions of Microbiomes with Bias Correction (ANCOM-BC) is an implemented DA method that overcomes the issue of bias introduced by differences in sampling fractions across samples(5). Outlier zeros, identified by finding outliers in the distribution of taxon counts within each sample grouping, were ignored during DA analysis, and replaced with NA. Structural zeros, taxa that were absent in one grouping, but present in the other, were ignored during data analysis and automatically called as differentially abundant. The ANCOM-BC algorithm iterates until convergence (100) with a specified tolerance level (1e-5). Results are controlled for a significance level (0.05) and global contrasts evaluated. FDR was applied. A taxon was called as differentially abundant if the number of corrected *P*-values reaching nominal significance for that taxon was greater than 90% of the maximum possible number of significant comparisons.

ALDEx2. We supplied the Phyloseq object to the ALDEx2 (ANOVA-Like Differential Expression version 2) function, which generated Monte Carlo samples of Dirichlet distributions for each sample, using a uniform prior performed relative log expression transformation of each realization. Wilcoxon tests were then performed on the transformed realizations. Finally, the function returned the expected Benjamini-Hochberg FDR-corrected *P*-value for each feature, based on the results of the difference across Monte Carlo samples.

Data availability

Raw sequencing data for each study can be accessed as described in the manuscript. Raw processed ASV tables can be accessed in GitHub space, available at http://github.com/PietropaoliLab. All other relevant data supporting findings of this study are available in the present manuscript and its Supplemental files, or upon request to the corresponding authors.

Supplemental acknowledgements

Oral DISeases and SYstemic interactions study group (ODISSY group), L'Aquila, Italy; founders: Davide Pietropaoli, Rita Del Pinto; collaborators: Claudio Ferri, Mario Giannoni, Annalisa Monaco, Eleonora Ortu, Serena Altamura.

Supplemental Data Figure Legends

Supplemental Figure 1. **Flow chart depicting data reduction approach.** After application of inclusion and exclusion criteria, a total of seven BioProjects were included in the analysis. NCBI, National Center for Biotechnology Information; SRA, Sequence Read Archive.

Supplemental Figure 2. Analysis of confounders reveal a major role for study heterogeneity and sampling sites in explaining microbial variance. **A**, Using a generalized linear model (GLM), the association between Log_{10} normalized phyla abundance and selected metadata (sex, age, smoking, sampling site, study, and library size) was tested. **B,** Microbial variance explained by sex was plotted against variance explained by the two major putative confounding factors in the analyses, namely the factors ‘study’ and ‘sampling site’. This analysis revealed the factors ‘study’ and ‘sampling site’ to have a predominant impact on microbial composition regarding variance explained by sex. Lib.size, library size.

Supplemental Figure 3. No evidence of sex-specific enrichment in oral microbiome of periodontally-healthy individuals. **A**, In periodontally-healthy individuals, there is no evidence of differential sex-based enrichment in genera at the study level, by Welch’s t-test with FDR applied. **B**, Heat map showing Log_{10} mean difference between sexes at phylum level across sampling sites (i.e., saliva, (dental) plaque and subgingival (plaque) in periodontally-healthy individuals, with no evidence of sex-specific significant enrichment *(right*). Phyla mean difference is colored by sex, with blue and pink indicating enrichment in male (M) and female (F) patients, respectively, and white indicating no difference between sexes using Welch’s t-test with FDR.

Supplemental Figure 4. **Circular plots showing level of agreement between methodologies for identification of sexually-dimorphic oral microbiome composition in periodontally-healthy individuals and during periodontitis**. Site-specific assessment (i.e., saliva, dental plaque and subgingival plaque) by periodontal condition (i.e., healthy and periodontitis) and considering smoking status (i.e., non-smokers, *top* and smokers, *bottom*) was performed, using a combination of methods for DA analysis (Welch's test(6)), RNA-Seq based (DeSeq2(7), LEfSe(8), ANCOM-BC(5), and ALDEx2(9)) (see **Methods** and **Expanded Methods**). For each microorganism, the number of bars represents the number of methodologies for DA analysis yielding consistent results in terms of sexual dimorphism in abundance. The consensus level between multiple methodologies for DA analysis allowed us to assess the reliability and consistency of findings and provide more context to improve their interpretation, according to the literature(1).

Supplemental Figure 5. Same-sex, within-site comparisons in alpha diversity between periodontal conditions across smoking habits. For non-smokers, saliva composition indicates increased richness in females with periodontitis compared with healthy females (*left*), while subgingival microbiome shows increased richness in males with periodontitis compared with healthy males (*right*). Composition of dental plaque from healthy males is richer than that of males with periodontitis (*right*). ASV, amplicon sequence variants; *P<0.05; **P<0.01, using the Wilcoxon test.

Supplemental Figure 6. **Scatter plot of Ab titers and microbial relative abundance at the genus level by sex in periodontally-healthy individuals and during periodontitis**.

Validation cohort was derived from the third National Health and Nutrition Examination Survey (NHANES III). Caucasian adults who underwent assessment of antibodies to 21 periodontal bacteria(10) in NHANES III (*N*=5825 with complete, validated periodontal exam) were paired 1:1 for sex, age, smoking (yes/no), and periodontal condition to a subset of individuals from the 7 included studies that underwent subgingival microbial sampling. Subgingival site was chosen for its increased exposure to underlying mucosal immune compartment compared to other oral sites (saliva, dental plaque)(11). Ab relative abundance at genus level was calculated (see **Supplemental Table 4**). A positive, female-specific correlation between genus-ranked Ab relative abundance and microbial abundance at the same taxonomic level is observed in individuals with periodontitis, while no evidence of sexual dimorphism in immune activation towards subgingival bacteria was found in periodontally-healthy conditions; calculated by Pearson's correlation coefficient.

References

1. Nearing JT, et al. Microbiome differential abundance methods produce different results across 38 datasets. *Nat Commun*. 2022;13(1):342.

2. Lozupone CA, et al. Diversity, stability and resilience of the human gut microbiota. *Nature*. 2012;489(7415):220–230.

3. yang. *yiluheihei/microbiomeMarker: microbiomeMarker 0.0.1*. Zenodo; 2020.

4. Cao Y, et al. microbiomeMarker: an R/Bioconductor package for microbiome marker identification and visualization. *Bioinformatics*. 2022;38(16):4027–4029.

5. Lin H, Peddada SD. Analysis of compositions of microbiomes with bias correction. *Nat Commun*. 2020;11(1):3514.

6. Welch BL. The generalisation of student's problems when several different population variances are involved. *Biometrika*. 1947;34(1-2):28–35.

7. Love MI, Huber W, Anders S. Moderated estimation of fold change and dispersion for RNA-seq data with DESeq2. *Genome Biol*. 2014;15(12):550.

8. Segata N, et al. Metagenomic biomarker discovery and explanation. *Genome Biol*. 2011;12(6):R60.

9. Fernandes AD, et al. Unifying the analysis of high-throughput sequencing datasets: characterizing RNA-seq, 16S rRNA gene sequencing and selective growth experiments by compositional data analysis. *Microbiome*. 2014;2:15.

10. Dye BA, et al. Serum antibodies to periodontal bacteria as diagnostic markers of periodontitis. *J Periodontol*. 2009;80(4):634–647.

11. Chhibber-Goel J, et al. Linkages between oral commensal bacteria and atherosclerotic plaques in coronary artery disease patients. *NPJ Biofilms Microbiomes*. 2016;2:7.

Supplemental Table 1. Characteristics of studies included in meta-analysis

BioProject	16s Region	Sampling site	Reads	Truncation length, left	Truncation length, right
PRJEB6047	V3	supragingival and subgingival plaque	F, R	150	140
PRJNA321534	V4	saliva and subgingival plaque	F	250	-
PRJNA324274	V4	subgingival plaque	F, R	250	240
PRJNA477241	V3-V4	subgingival plaque	F, R	280	220
PRJNA773202	V3-V4	subgingival plaque	F, R	280	220
PRJNA774299	V3-V4	saliva	F, R	280	220
PRJNA774981	V3-V4	saliva	F, R	280	220

F, forward; R, reverse

Supplemental Table 2. Propensity Score Matching (PSM) by sex of individuals with periodontitis from included studies (*N*=422)

	Level	Periodontitis females	Periodontitis males	*P*-value
N		211	211	
**Sex (%)**	female	211 (100.0)	0 (0.0)	<0.001
**Age, Yrs (mean (SD))**		50.69 (12.60)	50.69 (13.79)	0.997
**Smoking (%)**	yes	39 (31.5)	56 (45.2)	0.037
**Race (%)**	Caucasian	54 (100.0)	63 (100.0)	NA
**Site (%)**	saliva	45 (21.3)	56 (26.5)	0.445
	supragingival plaque	94 (44.5)	86 (40.8)	
	subgingival plaque	72 (34.1)	69 (32.7)	
**Geo (%)**	Brazil:Guarulhos	39 (18.5)	36 (17.1)	0.851
	Canada	14 (6.6)	12 (5.7)	
	China: Shanghai	34 (16.1)	36 (17.1)	
	not applicable	70 (33.2)	64 (30.3)	
	Portugal: OPORTO	5 (2.4)	9 (4.3)	
	Spain: SANTIAGO	49 (23.2)	54 (25.6)	
**BioProject (%)**	PRJEB6047	14 (6.6)	12 (5.7)	0.484
	PRJNA321534	70 (33.2)	64 (30.3)	
	PRJNA324274	39 (18.5)	36 (17.1)	
	PRJNA477241	34 (16.1)	36 (17.1)	
	PRJNA773202	31 (14.7)	27 (12.8)	
	PRJNA774299	8 (3.8)	19 (9.0)	
	PRJNA774981	15 (7.1)	17 (8.1)	

Supplemental Table 3. PSM by sex of periodontally-healthy individuals from included studies (*N*=148)

	Level	Healthy females	Healthy males	*P*-value
N		74	74	
**Sex (%)**	female	74 (100.0)	0 (0.0)	<0.001
**Age, Yrs (mean (SD))**		41.15 (12.48)	40.05 (12.58)	0.596
**Smoking (%)**	yes	15 (25.9)	15 (21.7)	0.737
**Race (%)**	Caucasian	45 (100.0)	44 (100.0)	NA
**Site (%)**	saliva	23 (31.1)	30 (40.5)	0.236
	supragingival plaque	17 (23.0)	20 (27.0)	
	subgingival plaque	34 (45.9)	24 (32.4)	
**Geo (%)**	Canada	16 (21.6)	5 (6.8)	<0.001
	not applicable	13 (17.6)	25 (33.8)	
	Portugal: OPORTO	12 (16.2)	26 (35.1)	
	Spain: SANTIAGO	33 (44.6)	18 (24.3)	
**BioProject (%)**	PRJEB6047	16 (21.6)	5 (6.8)	0.004
	PRJNA321534	13 (17.6)	25 (33.8)	
	PRJNA773202	26 (35.1)	21 (28.4)	
	PRJNA774299	5 (6.8)	14 (18.9)	
	PRJNA774981	14 (18.9)	9 (12.2)	

Supplemental Table 4. Genus-level taxonomic classification of antibodies to 21 periodontal bacteria assessed in NHANES III

Dataframe name	Stains	Kingdom	Phylum	Class	Order	Family	Genus	Reference (NCBI Link)
P. gingivalis	ATCC #33277, #53978	Bacteria	Bacteroidetes	Bacteroidia	Bacteroidales	Porphyromonadaceae	Porphyromonas	https://www.arb-silva.de/browser/ssu-138.1/AB035455
P. intermedia	ATCC #25611	Bacteria	Bacteroidetes	Bacteroidia	Bacteroidales	Prevotellaceae	Prevotella	https://www.arb-silva.de/browser/ssu-138.1/AB547686
P. nigrescens	ATCC #33563	Bacteria	Bacteroidetes	Bacteroidia	Bacteroidales	Prevotellaceae	Prevotella	https://www.arb-silva.de/browser/ssu-138.1/AB547696
T. forsythia	ATCC #43037	Bacteria	Bacteroidetes	Bacteroidia	Bacteroidales	Tannerellaceae	Tannerella	https://www.arb-silva.de/browser/ssu-138.1/AB035460
*A. actino-mix**	ATCC #43718, #29523, #33384	Bacteria	Proteobacteria	Gamma proteobacteria	Enterobacterales	Pasteurellaceae	Aggregatibacter	https://www.arb-silva.de/browser/ssu-138.1/AB512007
F. nucleatum	ATCC #10953	Bacteria	Fusobacteriota	Fusobacteriia	Fusobacteriales	Fusobacteriaceae	Fusobacterium	https://www.arb-silva.de/browser/ssu-138.1/AB514450
S. oralis	ATCC #35037	Bacteria	Firmicutes	Bacilli	Lactobacillales	Streptococcaceae	Streptococcus	https://www.arb-silva.de/browser/ssu-138.1/AB355617
M. micros	ATCC #33270	Bacteria	Firmicutes	Clostridia	Peptostreptococcales-Tissierellales	Family XI	Parvimonas	https://www.arb-silva.de/browser/ssu-138.1/AB729072
C. rectus	ATCC #33238	Bacteria	Campylo-bacterota	Campylo bacteria	Campylobacterales	Campylobacteraceae	Campylobacter	https://www.arb-silva.de/browser/ssu-138.1/AB595133

E. corrodens	ATCC #23834	Bacteria	Proteobacteria	Gamma proteobacteria	Burkholderiales	Neisseriaceae	Eikenella	https://www.arb-silva.de/browser/ssu-138.1/AB525415
E. nodatum	ATCC #33099	Bacteria	Firmicutes	Clostridia	Peptostreptococcales-Tissierellales	Anaerovoracaceae	[Eubacterium] nodatum group	https://www.arb-silva.de/browser/ssu-138.1/AZKM01000010
S. intermedius	ATCC #27335	Bacteria	Firmicutes	Bacilli	Lactobacillales	Streptococcaceae	Streptococcus	https://www.arb-silva.de/browser/ssu-138.1/ATFK01000001
C. ochracea	ATCC #33624	Bacteria	Bacteroidota	Bacteroidia	Flavobacteriales	Flavobacteriaceae	Capnocytophaga	https://www.arb-silva.de/browser/ssu-138.1/AB671761
V. parvula	ATCC #10790	Bacteria	Firmicutes	Negativicutes	Veillonellales-Selenomonadales	Veillonellaceae	Veillonella	https://www.arb-silva.de/browser/ssu-138.1/AB538437
A. naeslundii	ATCC #49340	Bacteria	Actinobacteriota	Actinobacteria	Actinomycetales	Actinomycetaceae	Actinomyces	https://www.arb-silva.de/browser/ssu-138.1/AB062278
P. melaninogenica	ATCC #25845	Bacteria	Bacteroidota	Bacteroidia	Bacteroidales	Prevotellaceae	Prevotella_7	https://www.arb-silva.de/browser/ssu-138.1/AB547693
S. noxia	ATCC #43541	Bacteria	Firmicutes	Negativicutes	Veillonellales-Selenomonadales	Selenomonadaceae	Selenomonas	https://www.arb-silva.de/browser/ssu-138.1/AF287799
T. denticola	OMGS #3271	Bacteria	Spirochaetota	Spirochaetia	Spirochaetales	Spirochaetaceae	Treponema	https://www.arb-silva.de/browser/ssu-138.1/AB621358
S. mutans	ATCC #25175	Bacteria	Firmicutes	Bacilli	Lactobacillales	Streptococcaceae	Streptococcus	https://www.arb-silva.de/browser/ssu-138.1/AB294730

*For analysis, only aa was used. Other serotypes (a and b) were not used
**Antibodies to periodontal bacteria were Log_{10+1}-normalized, and antibody relative abundance was calculated at genus level

Supplemental Table 5. Script details of differential abundance methods used

DA method	Script Settings
Welch's test	norm = "TSS",<br>p_adjust = "fdr"
LEfSe	norm = "TSS", # normalized the data using total sum scaling<br># kw_cutoff = 0.05, # Then it performed a wc<br>wilcoxon_cutoff = 0.05,<br>lda_cutoff = 2 # threshold score of 2.0 (default)
DESeq2	norm = "RLE", # relative log expression<br>sfType = "poscounts",<br>fitType='local',<br>contrast = c("male", "female"),<br>p_adjust = "fdr",
ANCOM-BC	p_adj_method = "fdr",<br>zero_cut = 0.90, # by default prevalence filter of 10% is applied<br>lib_cut = 0,<br>struc_zero = TRUE,<br>neg_lb = TRUE,<br>tol = 1e-5,<br>max_iter = 100,<br>conserve = TRUE,<br>alpha = 0.05,<br>global = TRUE
ALDEX2	transform = "identity", # Raw reads is recommended from the ALDEx2 paper<br>norm = "RLE", #relative log expression,<br>p_adjust = "fdr"

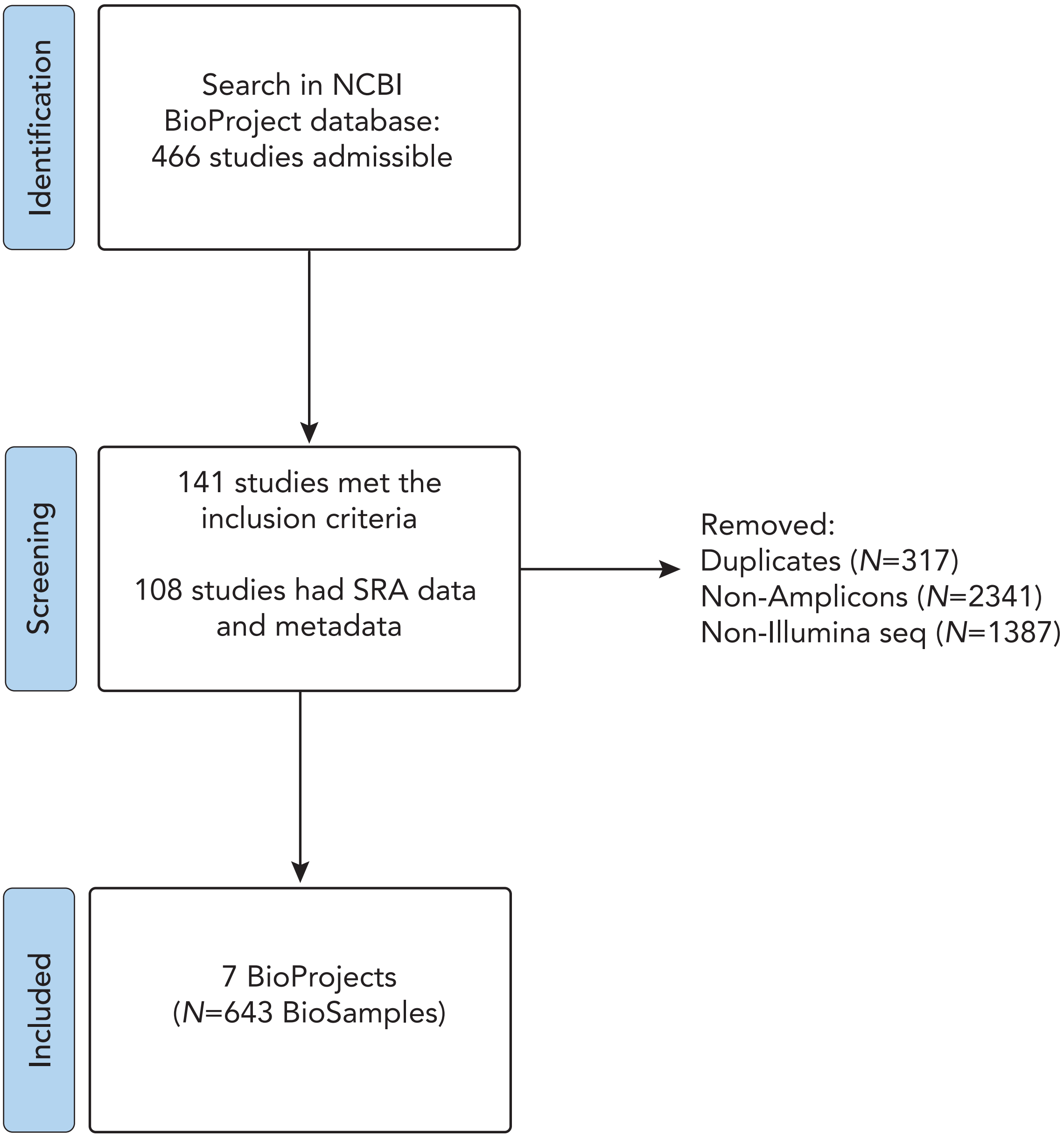

Supplemental Figure 1. Flow chart depicting data reduction approach.
After application of inclusion and exclusion criteria, a total of seven BioProjects were included in the analysis. NCBI, National Center for Biotechnology Information; SRA, Sequence Read Archive.

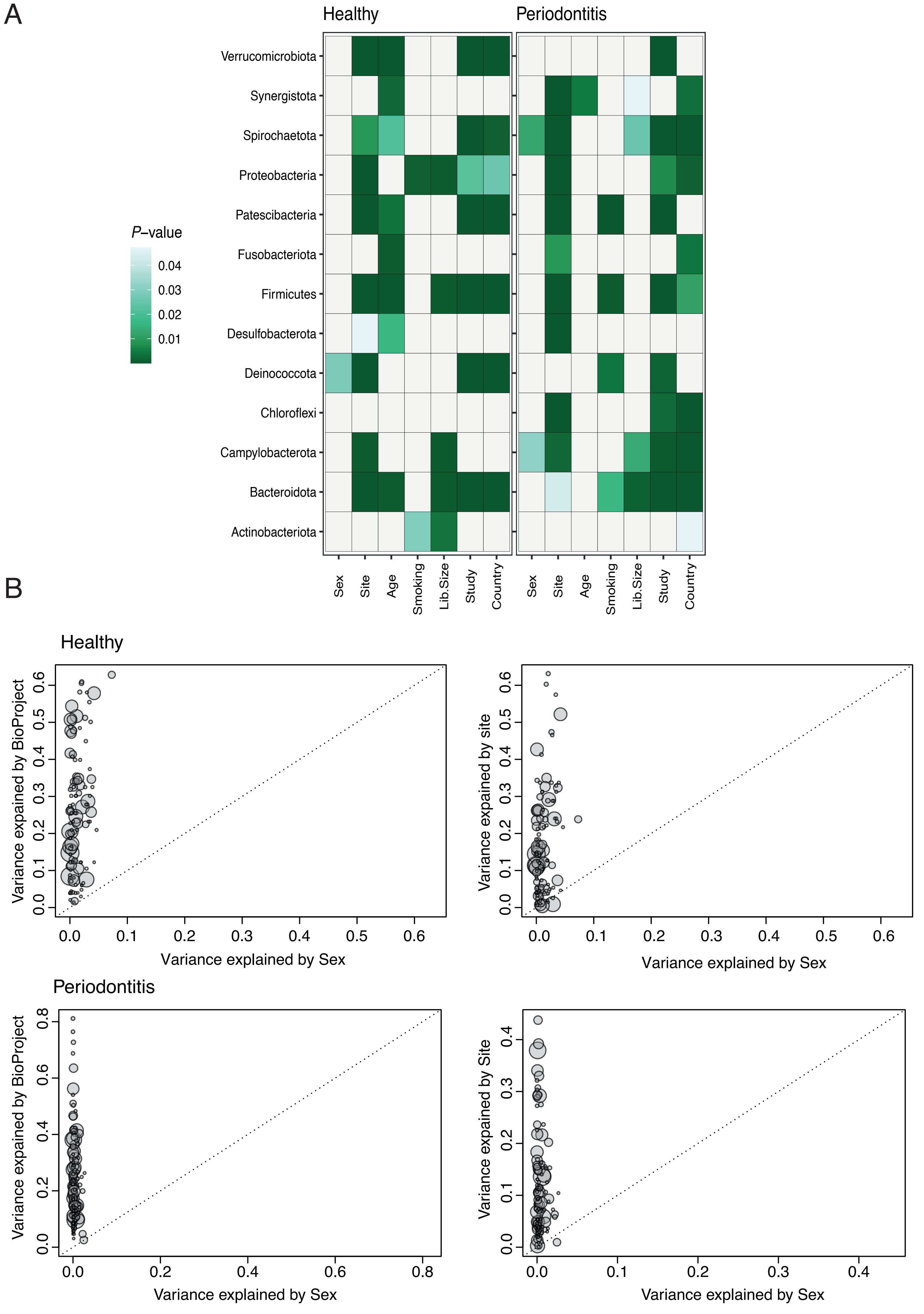

A
Healthy
Periodontitis
Verrucomicrobiota
Synergistota
Spirochaetota
Proteobacteria
Patescibacteria
Fusobacteriota
Firmicutes
Desulfobacterota
Deinococcota
Chloroflexi
Campylobacterota
Bacteroidota
Actinobacteriota
P–value
0.04
0.03
0.02
0.01
Sex
Site
Age
Smoking
Lib.Size
Study
Country
B
Healthy
Variance expained by BioProject
Variance explained by Sex
Variance expained by site
Periodontitis
Variance expained by BioProject
Variance expained by Site

Supplemental Figure 2. Analysis of confounders reveal a major role for study heterogeneity and sampling sites in explaining microbial variance.
A, Using a generalized linear model (GLM), the association between Log_{10} normalized phyla abundance and selected metadata (sex, age, smoking, sampling site, study, and library size) was tested. **B**, Microbial variance explained by sex was plotted against variance explained by the two major putative confounding factors in the analyses, namely the factors 'study' and 'sampling site'. This analysis revealed the factors 'study' and 'sampling site' to have a predominant impact on microbial composition regarding variance explained by sex. Lib.size, library size.

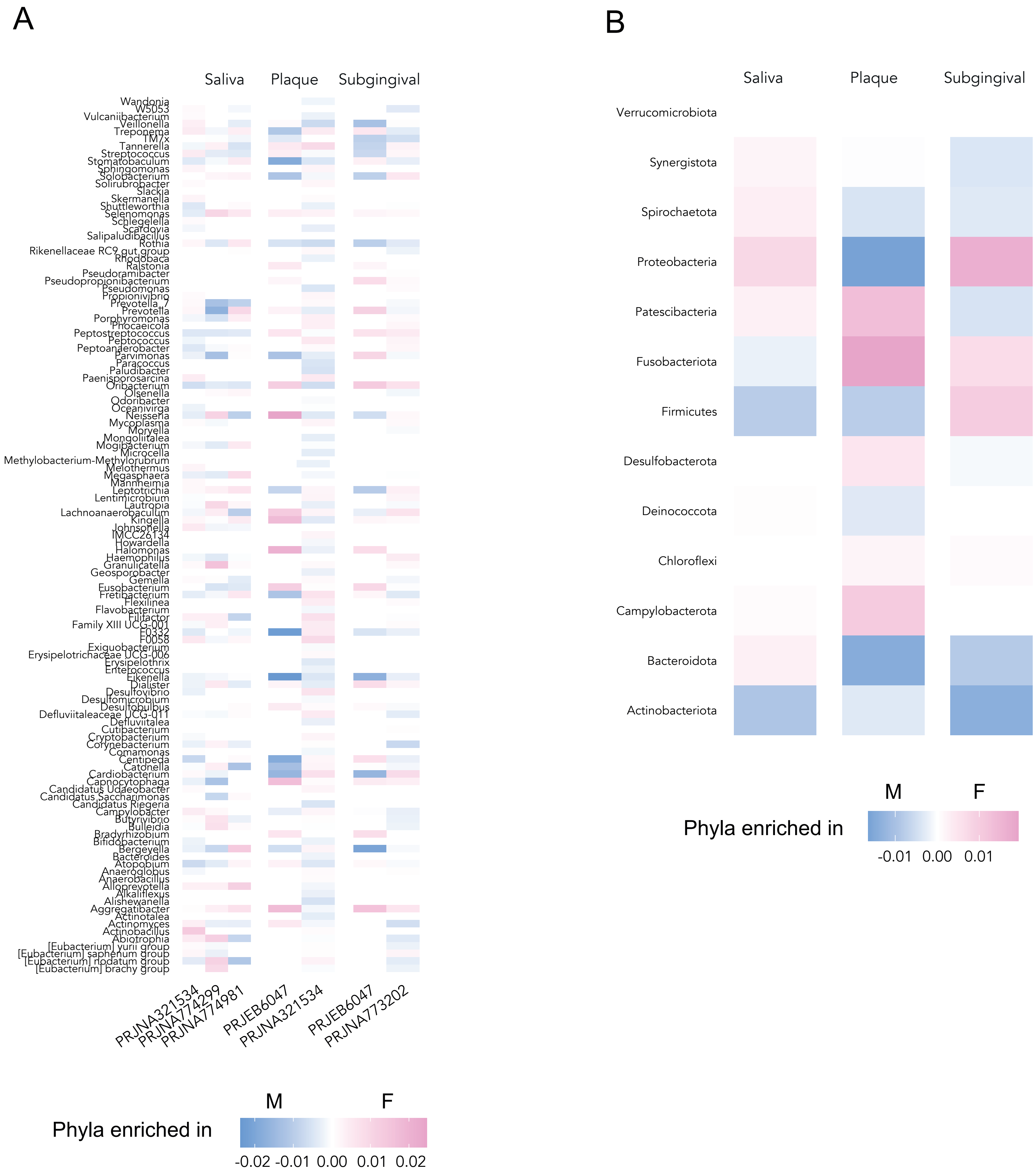

Supplemental Figure 3. No evidence of sex-specific enrichment in oral microbiome of periodontally-healthy individuals.
A, In periodontally-healthy individuals, there is no evidence of differential sex-based enrichment in genera at the study level, by Welch's t-test with FDR applied. **B**, Heat map showing Log_{10} mean difference between sexes at phylum level across sampling sites (i.e., saliva, (dental) plaque and subgingival (plaque) in periodontally-healthy individuals, with no evidence of sex-specific significant enrichment (*right*). Phyla mean difference is colored by sex, with blue and pink indicating enrichment in male (M) and female (F) patients, respectively, and white indicating no difference between sexes using Welch's t-test with FDR.

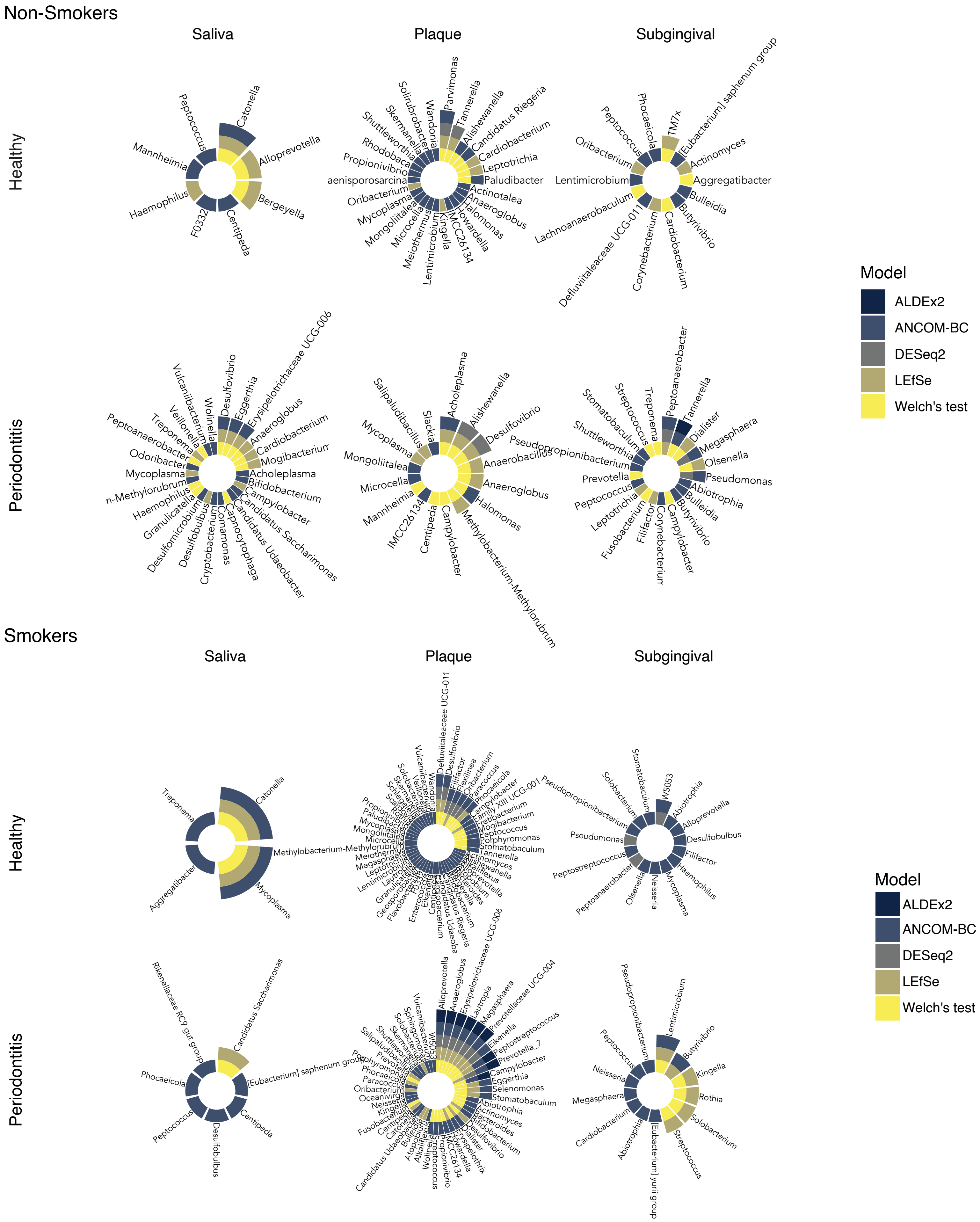

Supplemental Figure 4. Circular plots showing level of agreement between methodologies for identification of sexually-dimorphic oral microbiome composition in periodontally-healthy individuals and during periodontitis.

Site-specific assessment (i.e., saliva, dental plaque and subgingival plaque) by periodontal condition (i.e., healthy and periodontitis) and considering smoking status (i.e., non-smokers, *top* and smokers, *bottom*) was performed, using a combination of methods for DA analysis (Welch's test(6)), RNA-Seq based (DeSeq2(7), LEfSe(8), ANCOM-BC(5), and ALDEx2(9)) (see **Methods** and **Expanded Methods**). For each microorganism, the number of bars represents the number of methodologies for DA analysis yielding consistent results in terms of sexual dimorphism in abundance. The consensus level between multiple methodologies for DA analysis allowed us to assess the reliability and consistency of findings and provide more context to improve their interpretation, according to the literature(1).

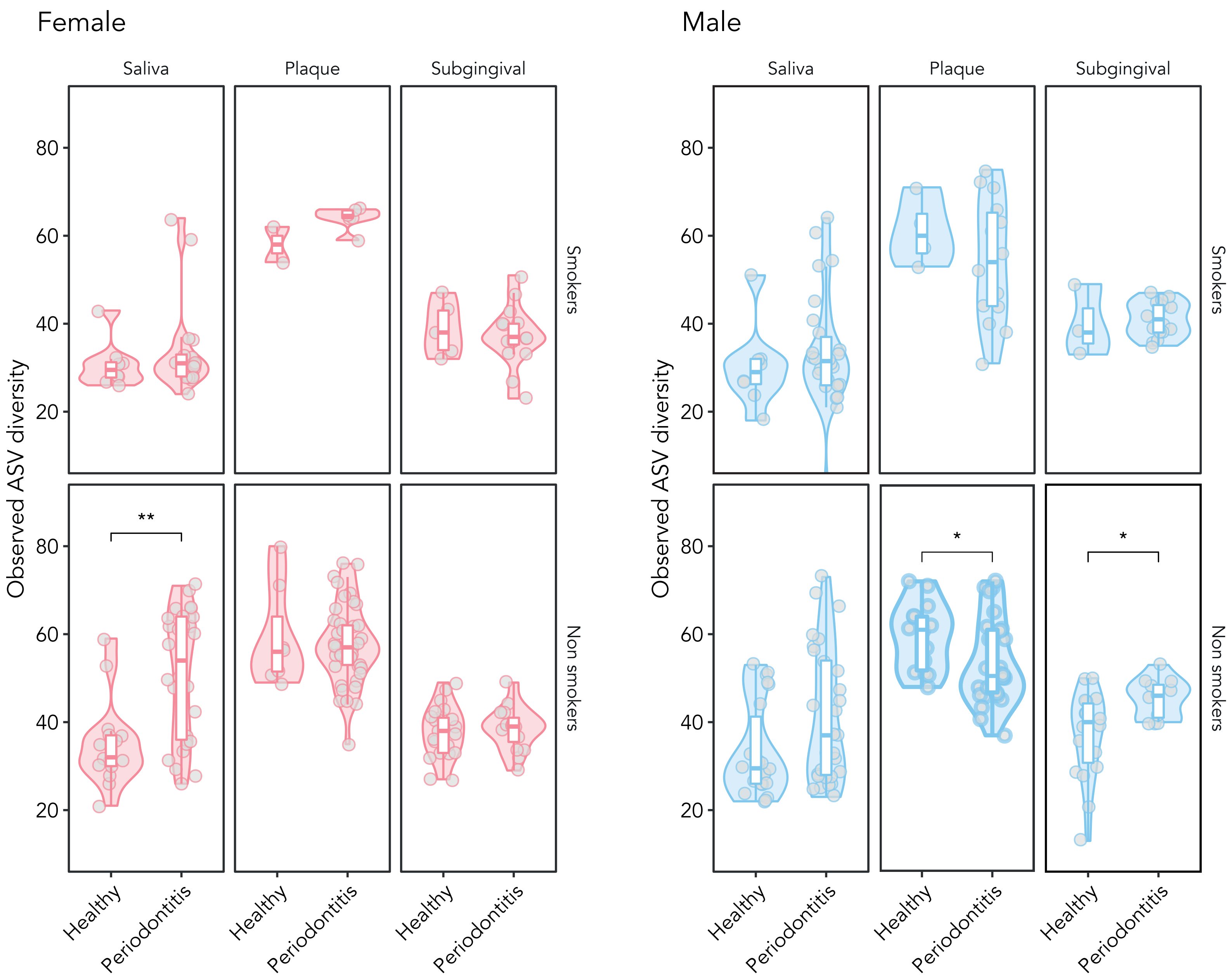

Supplemental Figure 5. Same-sex, within-site comparisons in alpha diversity between periodontal conditions across smoking habits.
For non-smokers, saliva composition indicates increased richness in females with periodontitis compared with healthy females (*left*), while subgingival microbiome shows increased richness in males with periodontitis compared with healthy males (*right*). Composition of dental plaque from healthy males is richer than that of males with periodontitis (*right*). ASV, amplicon sequence variants; *P<0.05; **P<0.01, using the Wilcoxon test.

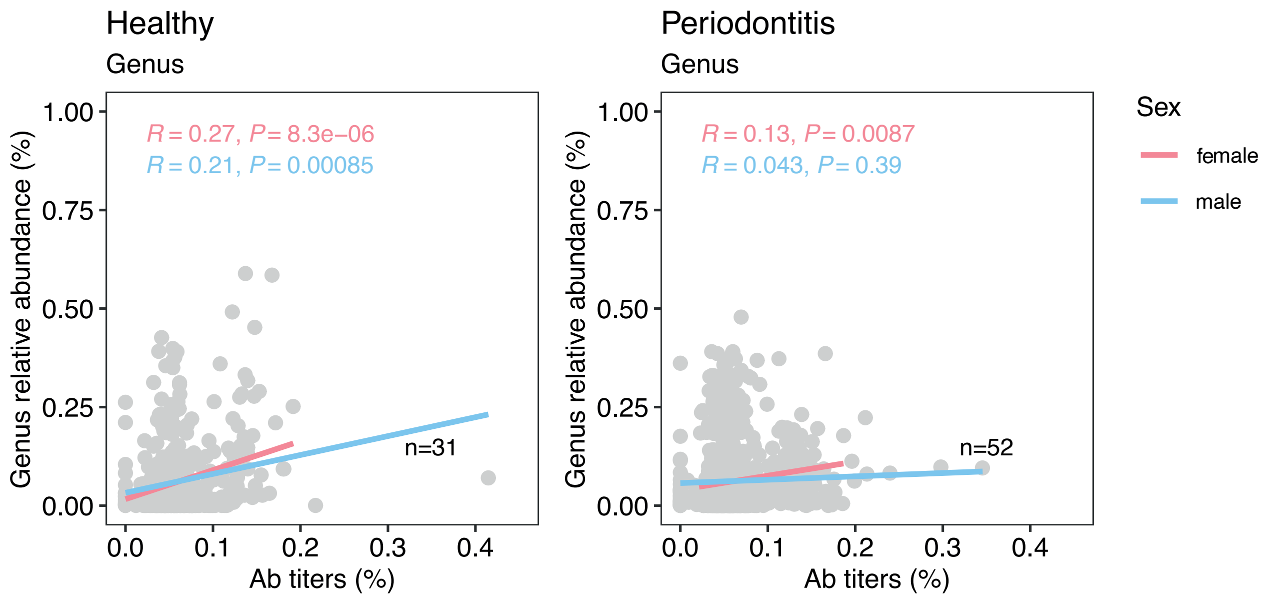

Supplemental Figure 6. Scatter plot of Ab titers and microbial relative abundance at the genus level by sex in periodontally-healthy individuals and during periodontitis.
Validation cohort was derived from the third National Health and Nutrition Examination Survey (NHANES III). Caucasian adults who underwent assessment of antibodies to 21 periodontal bacteria(10) in NHANES III (*N*=5825 with complete, validated periodontal exam) were paired 1:1 for sex, age, smoking (yes/no), and periodontal condition to a subset of individuals from the 7 included studies that underwent subgingival microbial sampling. Subgingival site was chosen for its increased exposure to underlying mucosal immune compartment compared to other oral sites (saliva, dental plaque)(11). Ab relative abundance at genus level was calculated (see **Supplemental Table 4**). A positive, female-specific correlation between genus-ranked Ab relative abundance and microbial abundance at the same taxonomic level is observed in individuals with periodontitis, while no evidence of sexual dimorphism in immune activation towards subgingival bacteria was found in periodontally-healthy conditions; calculated by Pearson's correlation coefficient.
